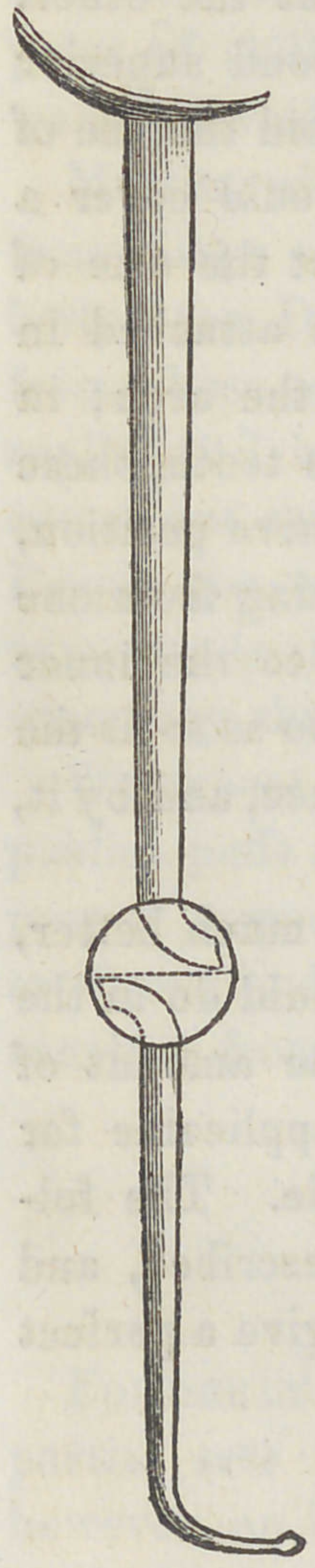# A Filtering Blow-Pipe

**Published:** 1859-09

**Authors:** Wm. Wm. Morgan

**Affiliations:** Somerset, Pa.


[For the Dental Register.

### A FILTERING BLOW-PIPE.

In consequence of the inconvenience suffered by the old method, and the loss usually sustained by the frequent breaking of the teeth, whilst undergoing the process of soldering, I was induced to devise some means whereby this might be obviated.

Accordingly, I constructed what I have styled a filtering blow-pipe, a rude drawing of which I forward with this description.

The pipe which I use is fifteen inches long; its larger orifice is one half inch in diameter; its terminus or smaller end, is tipped with the point of an ordinary gas burner furnished with a very small aperture.

There is a turned brass globe an inch and a quarter in diameter, situated one-third from the smaller end, which is put together by means of a screw; and in the more distant hemisphere of this globe, around the end of the pipe, there is nicely fitted a very fine sponge, which serves an excellent purpose as a filterer, taking up every particle of the *spray* caused by the breath, and thus removing a very common cause of fracture of the teeth most effectually.

Supposing the globe to be *glass*, the drawing gives you the pipe in perspective when put together, divested of the sponge. I have been using this pipe exclusively, for the last four years, and with a single exception, have not broken a tooth *old* or *new*.

WM. WM. MORGAN.

Somerset, Pa., July 14, 1859.

## Figures and Tables

**Figure f1:**